# Successful Treatment of Congenital Chylothorax by Early Use of Prednisolone: A Case Report

**DOI:** 10.7759/cureus.60628

**Published:** 2024-05-19

**Authors:** Kyoka Hirano, Koji Nakae, Manaka Matsunaga, Kentaro Ueno, Yasuhiro Okamoto

**Affiliations:** 1 Pediatrics, Kagoshima University Hospital, Kagoshima, JPN

**Keywords:** pleural effusion, fetal thoracentesis, drainage, chylothorax, prednisolone

## Abstract

Congenital chylothorax is the most common form of pleural effusion during the neonatal period; however, no treatment strategy exists. The pathogenesis and etiology of this disease are not fully understood; hence, several cases are difficult to treat. Some patients with chylothorax may not survive due to severe respiratory distress. Prednisolone (PSL) is sometimes used to treat congenital chylothorax but is rarely used in the early postnatal period. In this report, we describe a neonate with prenatal pleural effusion who was successfully treated with PSL from day one after requiring endotracheal intubation and ventilator management due to a postnatal diagnosis of chylothorax. The patient was extubated at four days of age, weaned from the ventilator at 10 days of age, and discharged home at 40 days of age after a total of 10 days of administration. Although the mechanism of action of PSL in chylothorax is unknown, and because it is a steroid, side effects such as gastrointestinal perforation and susceptibility to infection should be noted. The present case suggests the utility of early PSL administration for the treatment strategy of congenital chylothorax.

## Introduction

Congenital chylothorax is reported in one out of 2,000 neonatal intensive care unit admissions [1]. It may be associated with congenital lymphatic malformations, thoracic duct obstruction, thoracic duct hypoplasia, or genetic disorders such as Down syndrome, Turner's syndrome, and Noonan syndrome. Approximately 4.9% of fetuses diagnosed with Down syndrome experience pleural effusions [2]. If not treated properly, it can cause severe respiratory distress, metabolic disorders, immunodeficiency, and life-threatening nutritional complications [3]. To the best of our knowledge, evidence-based guidelines for the treatment of congenital chylothorax are lacking. Fasting milk, medium-chain triglycerides (MCT) milk, octreotide, and somatostatin are empirically used [4,5]. Reports have shown that prednisolone (PSL) is useful for congenital chylothorax; however, there are only a few reports on its early use. Herein, we report a rare case of congenital chylothorax that was successfully treated with the early use of PSL.

## Case presentation

**Figure 1:**
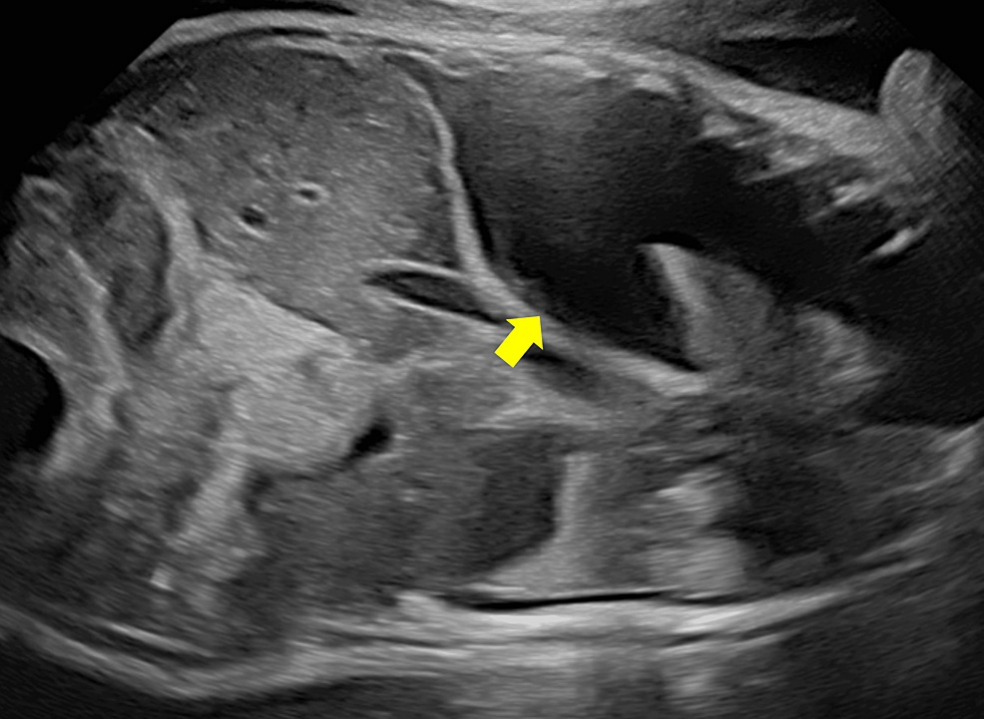
Fetal pleural effusion visualized on a fetal ultrasound image at 33 weeks, four days gestation (yellow arrow).

The patient was a 0-day-old male neonate with a birth weight of 2,188 g, born by cesarean section at 34 weeks and two days of gestation. Significant prenatal pleural effusion was observed in the fetus, and fetal thoracentesis was performed at 33 weeks and four days, draining 28 mL of fluid (Figure 1).

**Figure 2:**
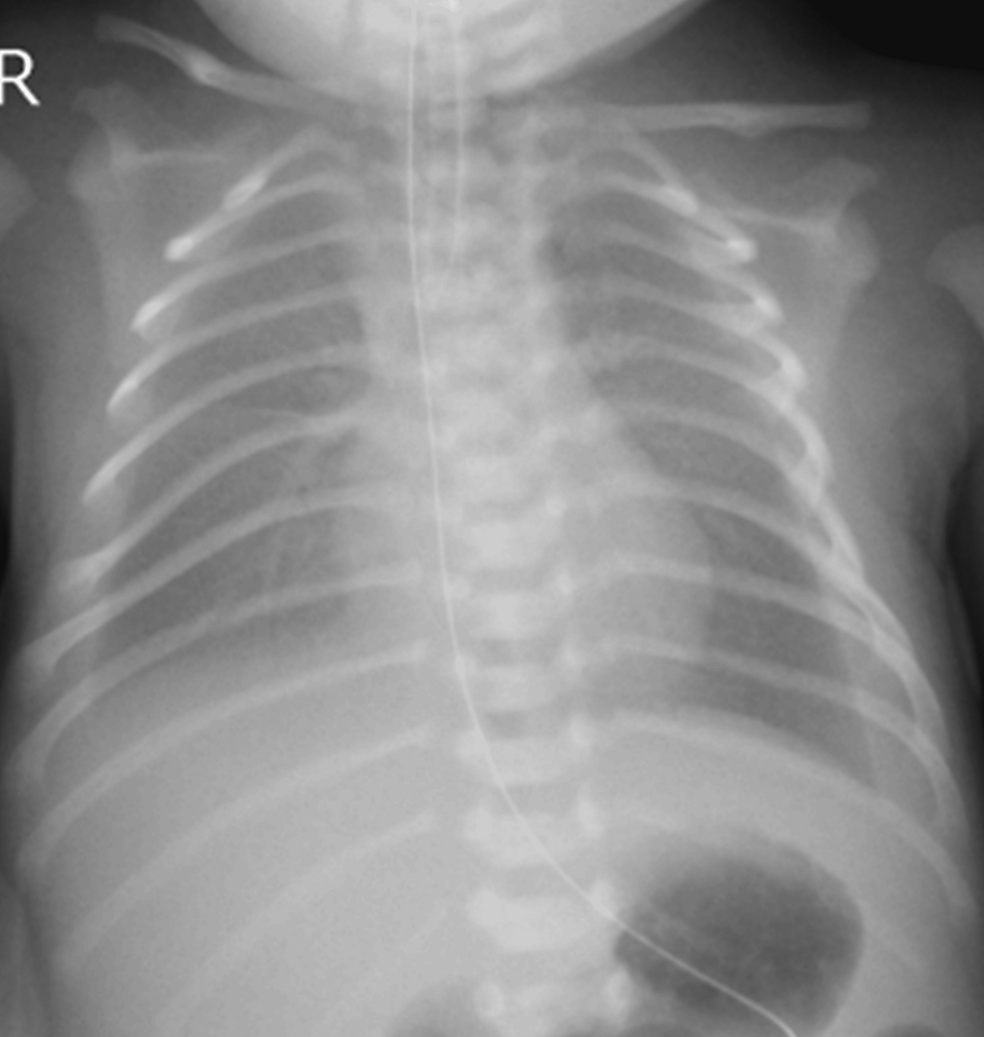
Chest radiograph on admission to the neonatal intensive care unit Bilateral pleural effusions with right interlobar pleural effusion are seen.

Furthermore, 70 mL of pleural effusion was drained immediately before the cesarean section. The lymphocyte count in the pleural fluid collected was 86.5%, indicating chylothorax. The patient had no spontaneous respiration immediately after birth and required neonatal resuscitation, tracheal intubation, and artificial respiration. Apgar scores at one and five minutes were seven and eight, respectively. Down syndrome was suspected based on facial appearance, including upward-slanting eyes, a flat nose, a low auricular position, and a short neck. The patient was admitted to the neonatal intensive care unit, where he was placed on a ventilator and started on total parenteral nutrition (TPN) while fasting. Radiography revealed bilateral pleural effusion, predominantly on the right side (Figure 2). Ultrasonography revealed no apparent congenital heart disease.

**Figure 3:**
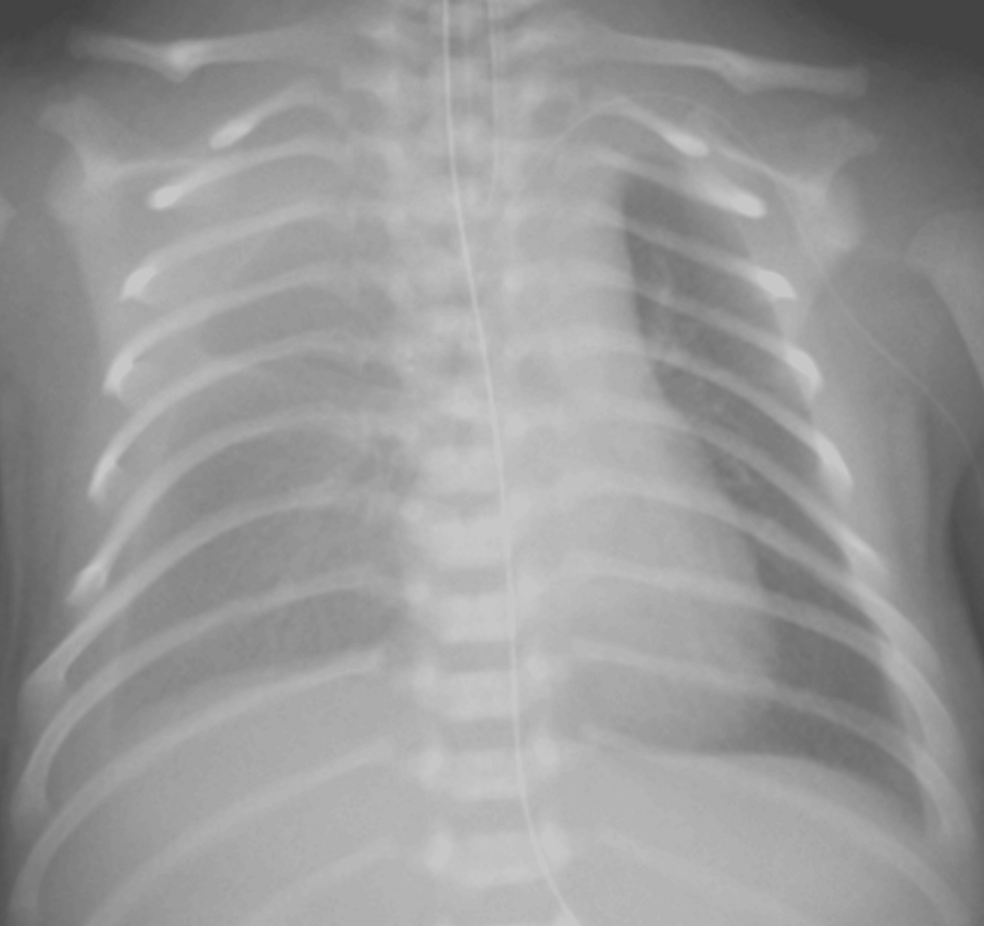
Chest radiograph on day one Increased right pleural effusion is seen.

As the pleural effusion increased on day one (Figure 3), continuous thoracic drainage was performed. Since the pleural fluid cell count was 2,524/μL and the lymphocyte ratio was 94.0%, the patient was diagnosed with chylothorax.

**Figure 4:**
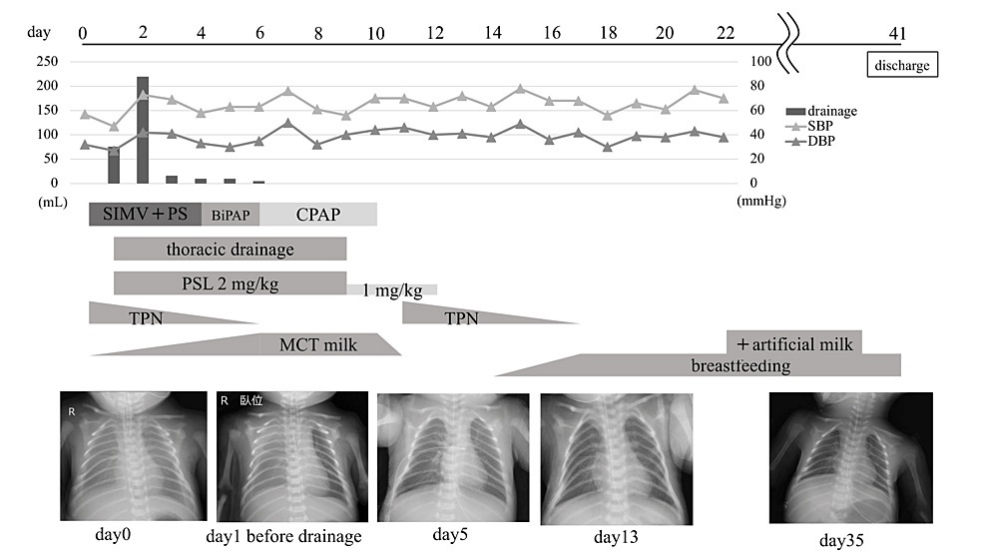
Clinical course BiPAP: biphasic positive airway pressure; CPAP: continuous positive airway pressure; DBP: diastolic blood pressure; PS: pressure support; PSL: prednisolone; SBP: systolic blood pressure; SIMV: synchronized intermittent mandatory ventilation; TPN: total parenteral nutrition; MCT: medium chain triglycerides

With the clinical course of pleural effusion beginning in the fetal period and assuming that the chylothorax was refractory, PSL (2 mg/kg/day) and enteral feeding with MCT milk were initiated under aggressive treatment. Subsequently, the pleural effusion gradually decreased. Extubation was performed at four days of life, and after extubation, the patient was placed on non-invasive positive pressure ventilation for 10 days. No adverse events due to PSL, such as hypertension or hyperglycemia, were observed. Thoracic drainage was terminated on day nine, and the PSL dose was reduced to 1 mg/kg/day. Subsequently, the PSL was discontinued on day 12 with no recurrence of pleural effusion. Although bloody gastric residuals appeared transiently on day 11, they were reduced by temporary fasting; breastfeeding was started on day 14, and TPN was terminated on day 17. On day 22, the patient was started on artificial milk and progressed without problems. The patient was feeding well and steadily gained weight; he was discharged on day 40 with a weight of 2,312 g (Figure 4).

**Figure 5:**
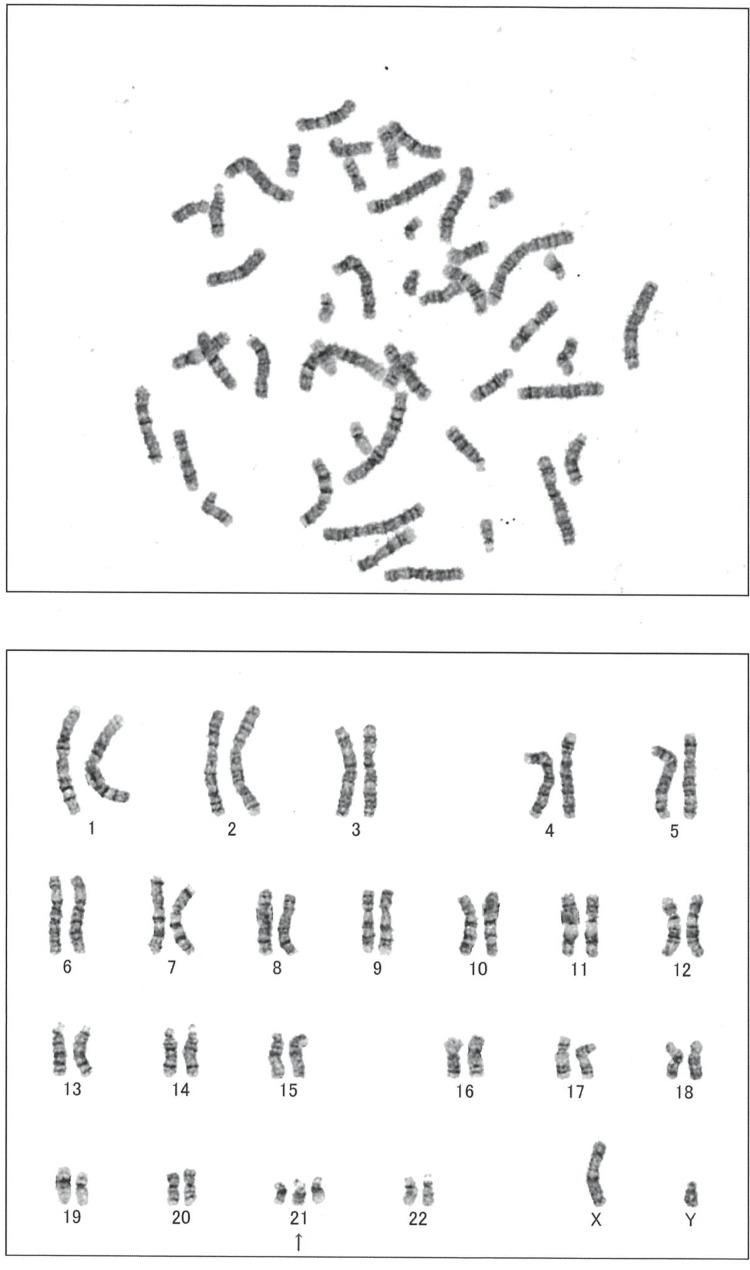
Karyotyping of the patient Chromosome test result indicates the patient's karyotype is 47,XY,+21.

In addition, the chromosome test result was 47,XY,+21, consistent with Down syndrome (Figure 5).

## Discussion

Chylothorax could result from either traumatic or non-traumatic causes. Non-traumatic chylothorax may be due to neoplasms, congenital, or idiopathic factors, while traumatic chylothorax is caused by surgical or non-surgical injuries [6]. Although chylothorax is a rare but life-threatening condition in newborns, treatment strategies are crucial because patients with chylothorax may require long-term hospitalization. However, there are no clear indications regarding the effectiveness, safety, or optimal treatment for the conservative management of chylothorax. Furthermore, no consistent recommendations for its management are available. The primary limitations include the lack of uniformity of regimens, the small number of patients, and wide variations in patient characteristics and clinical status between the studies. This patient had pleural effusion during the fetal period and required drainage and multidisciplinary treatment, including ventilator-assisted management after birth. Additionally, the patient was considered to have Down syndrome based on his facial appearance. Hence, we decided that active control of the chylothorax was necessary. Therefore, in addition to chest drainage, PSL was initiated on the first day of life.

Steroid treatment for chylothorax after cardiac surgery was first reported in 1987 by Rothman et al. [7]. Thereafter, a few reports indicated steroid drugs for treating congenital chylothorax; however, there are also reports that their effects are limited. Therefore, steroids are not an established treatment method. However, most reports have administered PSL after the onset of chylothorax, and few have demonstrated the efficacy of PSL in chylothorax early after birth. To the best of our knowledge, only 10 cases have been reported regarding the use of steroids for congenital chylothorax (Table 1) [8-15].

**Table 1:** Congenital chylothorax cases treated by steroids w: weeks; d: days; PSL: prednisolone; HDC: hydrocortisone; TPN: total parenteral nutrition; MCT: medium-chain triglycerides; -: not mentioned

Author(s), year	Gestational age	Birth weight (g)	Date when steroid was started	Steroid	Dose (mg/kg/day)	Other treatment	Outcome
Kazuhiro A et al., 2001 [8]	31w6d	1,710	Day 18	PSL	2	Drainage, TPN, MCT formula	Discharged and survived at seven months
Yoshitaka K et al., 2005 [9]	34w2d	3,458	Day 22	PSL	2	Drainage, TPN, MCT formula, pleurodesis	Discharged and survived
Fumihiro M et al., 2006 [10]	32w3d	1,791	Day 7, day 8	HDC PSL	2	Drainage, TPN	Discharged and survived on day 66
Tomotsune D et al., 2009 [11]	34w4d	2,453	Day 3	PSL	2	Fetal thoracentesis, drainage, TPN	Discharged and survived on day 75
Kaneko M et al., 2012 [12]	37w	2,898	-	HDC	-	Intrapleural minocycline, octreotide, MCT formula	Died on day 34
	31w	1,368	-	PSL	-	Intrapleural minocycline, octreotide, MCT formula	Discharged and survived
	34w	2,620	-	HDC	-	Intrapleural minocycline, octreotide, MCT formula	Discharged and survived
Hayashida K et al., 2019 [13]	30w3d	1,706	-	-	-	Drainage, TPN, octreotide, lymphovenous anastomosis	Alive at day 120
Fujino S et al., 2020 [14]	33w3d	2,114	-	PSL	2	Drainage, TPN, MCT formula, octreotide, coagulation factor XIII	Discharged and survived
Oikawa T et al., 2021 [15]	36w1d	3,426	-	PSL	-	Balloon angioplasty, drainage, TPN, MCT formula, octreotide	Discharged and survived on day 141

Prednisolone was selected in six cases and hydrocortisone in two cases. In one case, both PSL and hydrocortisone were used, whereas in the other case, the type of steroid used was not reported. For those cases disclosing the date of initiation, the earliest use of PSL was three days after birth; however, there have been no reports on the introduction of PSL from the first day after birth, as in this case.

The mechanism of action of PSL on the chylothorax is unclear. Prednisolone may increase sensitivity to endogenous vasoconstrictors in the cells of blood and lymph vessels, and the contraction of lymph vessels may inhibit chyle leakage. Furthermore, PSL may increase blood protein levels and plasma osmolality, which may cause water to move from the interstitium into the vasculature and reduce lymphatic flow [10].

When chylothorax is treated conservatively, breastfeeding is prohibited, and central venous nutrition is required for an extended period. If the effusion persists for a long time, hypoalbuminemia or hypogammaglobulinemia may occur; therefore, albumin and gammaglobulin preparations and fresh-frozen plasma are administered as required. Prolonged TPN can cause problems such as catheter infection, hepatic dysfunction, and atrophy of the intestinal mucosa. In addition, essential fatty acids may be deficient even with MCT milk. Therefore, the early introduction of PSL, as in this case, may help resolve the chylothorax. Prednisolone is a steroid, and one must be cautious about its side effects, such as gastrointestinal perforation and susceptibility to infection. Prednisolone was administered after weighing the risks and benefits of the steroids. The patient responded well, with no recurrence of chylothorax after PSL tapering, which was discontinued in 11 days.

Herein, we report a rare case of congenital chylothorax that improved after early postnatal PSL administration. Although further case series are needed, the early introduction of PSL therapy in addition to conservative therapy is considered an effective therapeutic strategy for the treatment of congenital chylothorax.

## Conclusions

Our case demonstrates the effectiveness of early administration of PSL for congenital chylothorax, even in neonates in the early postnatal period. Since standardized guidelines have not yet been established, careful consideration must be given to the management and treatment of neonates with congenital chylothorax. To improve the quality of life of the neonate, careful monitoring of the chylothorax is essential, and early introduction of PSL therapy in the event of deterioration is considered to be one of the most effective treatment strategies.
